# Enhanced Performance of Quantum Dot-Based Light-Emitting Diodes with Gold Nanoparticle-Doped Hole Injection Layer

**DOI:** 10.1186/s11671-016-1573-8

**Published:** 2016-08-24

**Authors:** Fei Chen, Qingli Lin, Hongzhe Wang, Lei Wang, Fengjuan Zhang, Zuliang Du, Huaibin Shen, Lin Song Li

**Affiliations:** 1 Key Laboratory for Special Functional Materials, Henan University, Kaifeng, 475004 People’s Republic of China; 2 Collaborative Innovation Center of Nano Functional Materials and Applications, Kaifeng, Henan Province People’s Republic of China

**Keywords:** Quantum dot, Light-emitting diodes, Au nanoparticle, Electroluminescence, Localized surface plasmon resonance

## Abstract

**Abstract:**

In this paper, the performance of quantum dot-based light-emitting diodes (QLEDs) comprising ZnCdSe/ZnS core-shell QDs as an emitting layer were enhanced by employing Au-doped poly(3,4-ethylenedioxythiophene)/polystyrene sulfonate (PEDOT:PSS) hole injection layer (HIL). By varying the concentration and dimension of Au nanoparticle (NP) dopants in PEDOT:PSS, the optimal devices were obtained with ~22-nm-sized Au NP dopant at the concentration with an optical density (OD) of 0.21. Highly bright green QLEDs with a maximum external quantum efficiency (EQE) of 8.2 % and a current efficiency of 29.1 cd/A exhibit 80 % improvement compared with devices without Au NP dopants. The improved performance may be attributed to the significant increase in the hole injection rate as a result of the introduction of Au NPs and the good matching between the resonance frequency of the localized surface plasmon resonance (LSPR) generated by the Au NPs and the emission band of QD layer, as well as the suppressed Auger recombination of QD layer due to the LSPR-induced near-field enhanced radiative recombination rate of excitons. These results are helpful for fabricating high-performance QD-based applications, such as full-color displays and solid-state lighting.

**Graphical Abstract:**

80 % enhancement of efficency of quantum dot-based light-emitting diodes with gold nanoparticle doped hole-injection-layer.
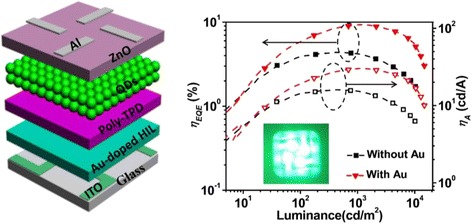

**Electronic supplementary material:**

The online version of this article (doi:10.1186/s11671-016-1573-8) contains supplementary material, which is available to authorized users.

## Background

Colloidal quantum dots (QDs) are promising light-emitting materials because of their color purity, color stability, and color tunability. Now, hybrid organic-QD light-emitting diodes (QLEDs), which consist of organic charge injection/transport layers and colloidal QD emitting layer, possess the advantages of both QDs and organic light-emitting diodes (OLEDs) including high efficiency, flexibility, and low processing cost [1–4]. As one of the most widely studied hole injection material in QLEDs, poly(3,4-ethylenedioxythiophene)/polystyrene sulfonate (PEDOT:PSS) has attracted great interest in both academic research and industry due to its easy deposition, low surface roughness, low cost, high transparency in the visible region, high stability in the oxidized state, aqueous solution processibility, and excellent thermal stability [5–9]. PEDOT:PSS has also been used as polymeric anode for capacitors or photodiodes and as buffer or hole injection/transport layer (HIL/HTL) in OLEDs, photovoltaic devices (PVs), and QLEDs [10–13]. For OLEDs and QLEDs, PEDOT:PSS plays a critical role in the balanced injection of electron and hole [14, 15]. However, the electrical conductivity of the PEDOT:PSS film is usually too low (usually below 1 S/cm) to meet the requirements of high-performance QLEDs. The low conductivity of PEDOT:PSS is one of the reasons for the charge injection imbalance. In recent years, many groups have tried many methods to improve the charge balance. Especially in 2014, Peng’s group inserted an extra insulating layer and sacrificed the electron injection to balance the carrier injection [16]. However, the improvement of hole injection would be a more efficient way to balance the injected carriers for the formation of excitons in QLEDs.

Many methods for improving the hole injection have reported, such as surface engineering [13], adjusting the devices architectures [16] and optimizing materials of carrier transport layer (CTL) [17] and the injection ability improvement of the HIL. For example, UV irradiation of PEDOT:PSS could increase the work function and thus enhance the hole injection efficiency of PEDOT:PSS. [8] In addition, the electrical conductivity of PEDOT:PSS film was also improved by adding dimethyl sulfoxide (DMSO) or doping sorbitol, grapheme, iron oxidant-hemin and Au-doped SWCNTs, etc [18–22]. These above methods have been applied to OLEDs, polymer light-emitting diodes (PLEDs), and organic photovoltaic devices (OPVs) with the improvements of the lower surface roughness of anode, higher work function, and electrical conductivity of HIL [23, 24]. Among the methods mentioned above, the metallic nanoparticles (such as Au, Ag, Cu) doped HIL attracts more attention, since these metallic nanoparticles have the advantages of superior optical properties such as localized surface plasmon resonance (LSPR) effect, energy transfer, metal enhanced fluorescence, and interface effect and are more suitable for large-area and scalable fabrication [25–33]. For example, the power conversion efficiency (PCE) of OPVs was improved by 20 % after blending Au NPs into the anodic buffer layer [34]. The PCE of the polymer photovoltaic cells (PSCs) could be improved by 22 % by incorporating PEG-capped Au NPs into PEDOT:PSS [35]. The enhanced performance of the PLEDs was achieved by embedding an ultra-thin Au NP layer on the cathode, resulting in enhanced luminous efficiency from 15.4 to 18.3 cd/A [36]. In addition, plasma-enhanced green QLEDs by incorporating solution processable Au NPs into devices demonstrated a significant enhancement of 116 % for both luminance and current efficiency [37].

These results spark the idea of using a simple device architecture to assess the effect of Au-doped PEDOT:PSS HIL on the performance of QLEDs. In this study, Au NPs with various concentrations indicated by the optical density (OD, from OD = 0.05 to 0.26, obtained from the absorption spectra) and sizes (from ~12 to ~26 nm) were doped into the PEDOT:PSS buffer layer and the electrical, optical, and morphological properties of QLEDs were evaluated and analyzed in details. The optimum device performance could be obtained when the Au NP dopants with a particle size and an OD value of ~22 nm and 0.21, respectively, were adopted. The maximum EQE of 8.2 % and a corresponding current efficiency of 29.1 cd/A show nearly 80 % enhancement compared with that of devices without Au NP dopants. The improved performance might be attributed to the significant increase in the hole injection, which benefits from the good conductivity of Au and balances the injected charge and produces more excitons within the emitting layer, and the matching between the resonance frequency of the LSPR generated by the Au NPs and the emission band of QDs layer, as well as the suppressed Auger recombination of QD layer due to the LSPR-induced near-field enhanced radiative recombination rate of excitons.

### Methods

#### Synthesis of ZnCdSe/ZnS Core-Shell QDs with Green Emission

The ZnCdSe/ZnS core-shell QDs and Au NPs were synthesized on the basis of previously reported methods [38, 39]. The synthetic procedures used in this work are described in detail in the Additional file 1.

#### Device Fabrication

QLEDs were fabricated on glass substrates pre-patterned with ITO with a sheet resistance of 20 Ω/sq. The ITO-coated glass substrates were sonicated sequentially in detergent, deionized water, acetone, and isopropanol, each for 15 min and followed by UV-ozone treatment in air for 15 min. The Au NP aqueous solutions (containing 12-, 16-, 22-, and 26-nm-sized Au NPs and OD values of 1.01, 1.04, 1.06, and 1.07, respectively) were centrifuged at 20,000 rpm, and then the precipitates were dispersed into the aqueous solution of PEDOT:PSS (AI 4083) under stirring with concentrations ranging from OD = 0.05 to 0.26. The Au NP-doped PEDOT:PSS aqueous solution was used to deposit HIL via spin-coating onto the top of ITO-covered substrates which were then transferred to N_2_-filled glovebox right after an annealing process at 140 °C for 15 min in air. Poly(4-butylphenyl-diphenylamine) (poly-TPD) (ADS254BE) was dissolved in chlorobenzene at a concentration of 8 mg/mL, which was then deposited onto the Au-doped PEDOT:PSS layer (3000 rpm for 30 s) and annealed at 150 °C for 30 min. In turn, ZnCdSe/ZnS core-shell QDs (18 mg/mL in toluene) and ZnO NPs (30 mg/mL in ethanol) were spin-coated onto the poly-TPD layer at different speeds and 3000 rpm for 30 s, respectively, followed by baking at 60 °C for 30 min. Al cathode that was patterned by a pre-prepared mask to form an active device area of 4 mm^2^ was consecutively deposited via thermal evaporation. The devices were then encapsulated with a cover glass fixed with low-permeation epoxy resin.

#### Characterization

Room temperature UV-vis absorption spectra were measured with an ultraviolet-visible-near-infrared spectrophotometer (mode PE Lambda 950), and the PL spectra were measured with a fluorescence spectrometer (mode JY HORIBA FluoroLog-3). PL quantum yields (QYs) were collected using an absolute PL QY measurement system (FLSP920) with an integrating sphere. Transient PL measurements were carried out using Edinburgh Instruments FL920 Spectrometer, and the excitation wavelength (hydrogen lamp as the excitation source) was 405 nm. The transmission electron microscopy (TEM) images of Au NPs were taken using a JEOL JEM-2100 electron microscope operating at 200 kV. Scanning electron microscopy (SEM) images were obtained using a Hitachi F-4800 FEG SEM. The morphology and topographic images including the root-mean-square (RMS) values with and without Au NPs in PEDOT:PSS HIL were analyzed by an atomic force microscope (AFM) (SPA-400). The current density-voltage-luminance characteristics and electroluminescence (EL) spectra of the QLEDs were measured by a programmable Keithley Model 2400 power supply and a Photo-Research PR735 spectrometer at room temperature under atmospheric conditions.

## Results and Discussion

**Fig. 1:**
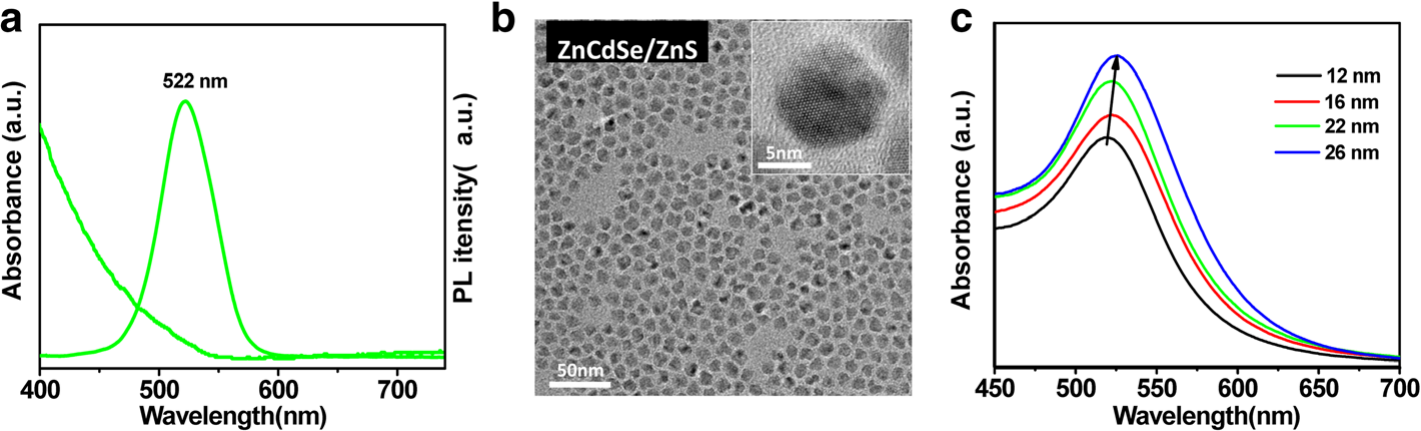
**a** UV-vis absorption and PL emission spectra of ZnCdSe/ZnS core-shell QDs. **b** TEM image of ZnCdSe/ZnS core-shell QDs and high-resolution TEM image of the QDs. **c** UV-vis absorption spectra of different-sized Au NPs in water

Figure 1a and Additional file 1: Figure S1 show the UV-vis absorption/PL characteristics of the as-prepared green ZnCdSe core and ZnCdSe/ZnS core-shell QDs dispersed in toluene and the increase of full width at half-maximum (FWHM) from 40 to 52 nm as the growth of shells. The absolute PL QY of ZnCdSe/ZnS core-shell QDs was measured to be up to 74 %. To further characterize the evolution of structures of ZnCdSe/ZnS core/shell QDs, their crystallographic properties were determined by XRD (Additional file 1: Figure S2). According to the XRD patterns, the peaks of ZnCdSe QDs lie between the bulk ZnSe and CdSe in a zinc blende structure. It is also clearly shown that the diffraction peaks shifted to that of the ZnS shell materials, indicating well-grown shells onto the ZnCdSe cores. TEM and high-resolution TEM (HRTEM) images of ZnCdSe cores and ZnCdSe/ZnS core/shell QDs are shown in Fig. 1b and Additional file 1: Figure S3. The core/shell QDs have a relatively uniform size distribution with an average diameter of 11 ± 1 nm after the shell coating process of the original core QDs with a mean diameter of 5.3 ± 0.8 nm as indicated by the TEM image. The corresponding HRTEM images of ZnCdSe cores and core/shell QDs are shown in Fig. 1b and Additional file 1: Figure S3 (insets), respectively. The HRTEM images of those QDs reveal high crystallinity with continuous lattice fringes throughout the whole particles and exhibit no diffraction contrast or interface between the core and shell. This implies that the growth of shells occurs in the regime of coherent epitaxy [38]. As shown in the HRTEM image of ZnCdSe core QDs, the (111) planes are separated by *d* = 0.33 nm, which is bigger than that of zinc blende ZnSe (*d* = 0.32 nm) and smaller than that of zinc blende CdSe (*d* = 0.35 nm). After the overcoating of ZnS, the (111) planes are separated by *d* = 0.31 nm, which is consistent with that of zinc blende ZnS, clearly demonstrating the well-coated shells on core QDs. The TEM images of different-sized Au NPs are shown in Additional file 1: Figure S4. The particle sizes measured for the samples shown in Additional file 1: Figure S4 are ~12, ~16, ~22, and ~26 nm, respectively. Figure 1c shows the UV-vis absorption spectra of different-sized Au NPs in water. With the dimension increase of Au NPs, the corresponding absorption peaks shift slightly towards the longer wavelength, which might be attributed to the dielectric constant that becomes size-dependent when particle sizes are much smaller than the wavelength of the exciting radiation [40, 41]. Furthermore, to investigate the variation of surface morphology of PEDOT:PSS film with and without Au NPs, atomic force microscopy (AFM) was performed to characterize the films with different Au NP concentrations. According to the AFM images shown in Additional file 1: Figure S5, the root-mean-square (RMS) roughness of the films fluctuate slightly for only 0.2 nm, indicating a negligible effect of the Au NP dopants on the surface roughness of PEDOT:PSS HIL films.

**Fig. 2:**
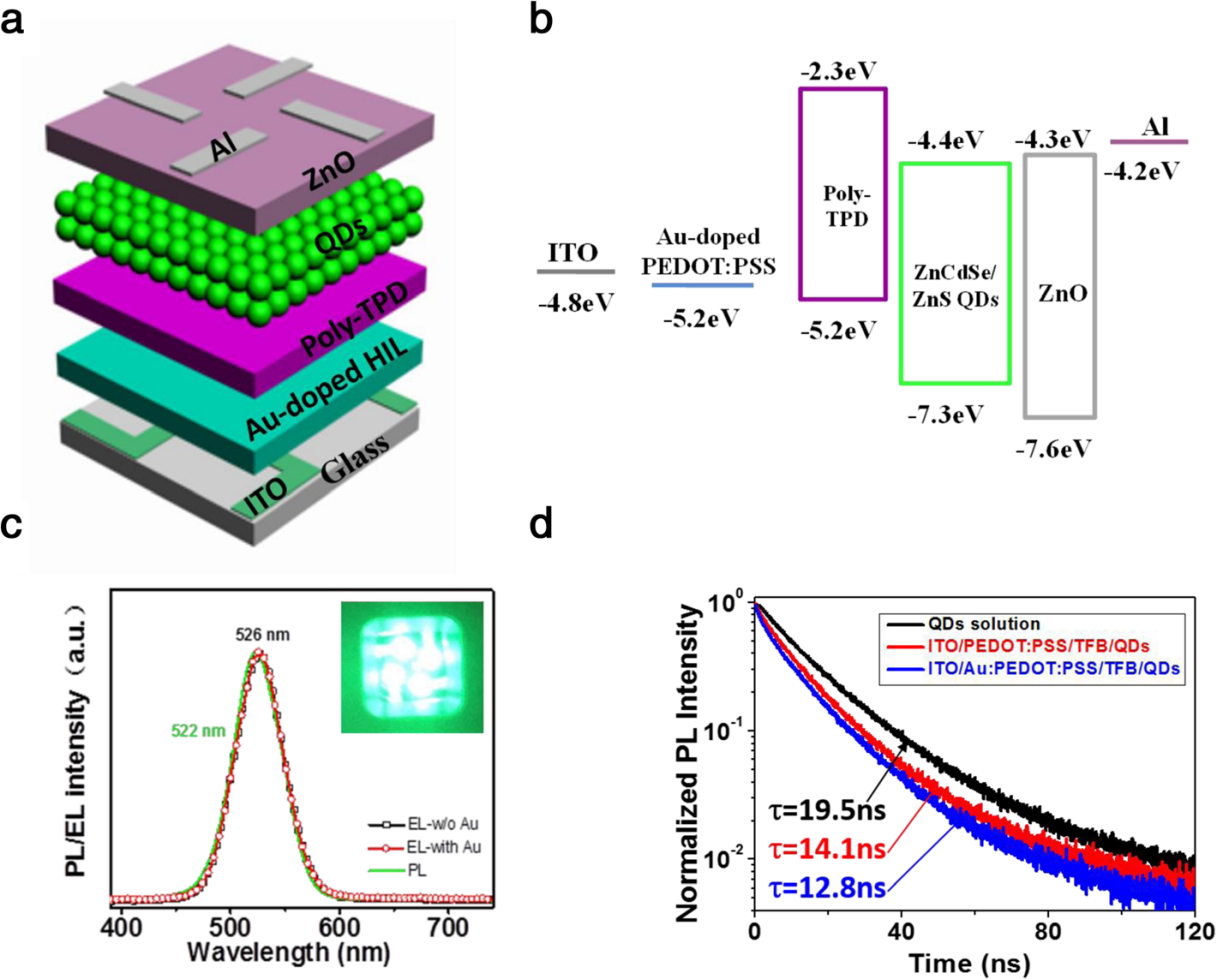
**a** Schematic illustration of all-solution-processed QLEDs with a multilayered structure, consisting of ITO/HIL/HTL/CdZnSe/ZnS core-shell QDs/ZnO NPs/Al. **b** Energy levels of multilayered structure. The values of energy level are cited from refs. [55–59]. **c** Normalized PL (*green line*) of QD solution and EL spectra of QLEDs for different hole injection materials (with and without Au NP-doped PEDOT:PSS). **d** PL decay curves of diluted toluene solution of ZnCdSe/ZnS core-shell QDs (*black line*) versus films of ITO/PEDOT:PSS/poly-TPD/QDs film (*red line*) and ITO/Au NP-doped PEDOT:PSS/poly-TPD/QDs film (*blue line*)

To evaluate the performance of QLEDs with the Au NP-doped PEDOT:PSS layer, all-solution-processed devices were fabricated, with a schematic structure illustration shown in Fig. 2a. The QLEDs with a multilayered structure consist of patterned ITO/HIL/poly-TPD/ZnCdSe/ZnS core-shell QDs/ZnO NPs/Al. All layers were sequentially spin-coated on a pre-patterned ITO glass substrate, except for Al cathode that was deposited by thermal vacuum deposition. Such device structure was adopted from our previous report with only a little modification of doping Au NPs into the PEDOT:PSS layer [13]. This device architecture enhances hole injection and slightly hinders electron injection. The energy levels of the multilayered structure are shown in Fig. 2b. ITO was used as the anode due to low resistivity, high work function, and transparency. The Au NP-doped PEDOT:PSS layer was spin-coated onto the ITO anode as buffer layer which plays a significant role in enhancing hole injection efficiency. Poly-TPD, with an excellent hole transport mobility, lowest unoccupied molecular orbital (LUMO) level of −2.3 eV, and highest occupied molecular orbital (HOMO) level of −5.2 eV, which matches well with the work function of the ITO/Au NP-doped PEDOT:PSS anode and contributes to the hole accumulation in the region of QDs layer, was used as HTL material. In addition, toluene, which is often used as solvent for good dispersion of QDs, is orthogonal to the solvent of poly-TPD. Thus, the poly-TPD layer can resist the damage during the subsequent spin-coating process of QD layer. As electron transport layer (ETL), ZnO can ensure the efficient electron injection as well as hole blocking due to the high electron mobility and valence band offset at the QD/ZnO interface, leading to an improved charge recombination efficiency. The EL spectra of devices with and without Au NPs and PL spectrum of QDs in solution are shown in Fig. 2c. The shape of EL spectrum of the optimal device matches well with the PL spectrum of QDs solution. There is no noticeable parasitic EL emission from the adjacent poly-TPD layer at ~430 nm, indicating that the major emission is originated from the ZnCdSe/ZnS core-shell QD layer. The EL peak (526 nm) is slightly red-shifted (4 nm) compared with the PL peak (522 nm) of QD solution due to the Förster resonant energy transfer (FRET) from smaller to larger dots within the QD layer [17, 42, 43]. Also, by comparing the EL spectra of Au NP-doped device with the one without Au NPs, the result indicates that the incorporation of Au NPs has no influence on the emission features of devices and the recombination region can still be confined in the QD layer. To further assess the effect of Au NPs on the properties of QD layer, time-resolved PL measurements were performed on diluted toluene solution of ZnCdSe/ZnS core-shell QDs, ITO/PEDOT:PSS/poly-TPD/QDs film, and ITO/Au NP-doped PEDOT:PSS/poly-TPD/QDs film, respectively. From Fig. 2d, we can see that the lifetime of QD solution reduced from 19.5 to 14.1 ns for ITO/PEDOT:PSS/poly-TPD/QDs film. This shortening (∼72.3 % remaining) is less pronounced than that of high-quality core-shell QDs synthesized by shell growth at a low temperature [44–46]. High-quality core/shell QDs synthesized using high temperature injection and lower temperature shell growth method usually possess long lifetime in liquid phase (10–30 ns). Their lifetime in the form of closely packed thin film become severely reduced as a result of the efficient FRET [44–48], typically retaining <40 %. Hence, the ZnCdSe/ZnS core-shell QDs with thicker composition gradient shells may act as effective spacers between interacting dipoles in neighboring QDs which should be highly advantageous in suppressing the FRET process, and such characteristics are expected to cater the fabrication of high-efficiency QLEDs. On the other hand, the average PL lifetime of ITO/PEDOT:PSS/poly-TPD/QDs film decreases from 14.1 to 12.8 ns after doping of Au NPs into HIL due to the LSPR induced by the Au NPs in HIL. It indicates that adding Au NPs into HIL shortens the lifetime of the excitons in the QD-emitting layer, enhances the radiative recombination rate of excitons, and thus reduces the Auger recombination caused by QDs charging effectively due to the decreased concentration of excitons in the emission layer [37]. Furthermore, we also collected the decay time of ZnCdSe/ZnS core-shell QD solution samples at different emission wavelengths, i.e*.*, 490, 522, and 565 nm (as shown in Additional file 1: Figure S6). There is only moderate decrease from 21.8 to 19.1 ns with increasing emission energy. These PL decay results indicate that the thicker gradient composition shells of ZnCdSe/ZnS core-shell QDs, which were synthesized by “low temperature injection and high temperature shell growth” method [23], can effectively reduce the FRET probability between the interacting QD dipoles.

**Fig. 3:**
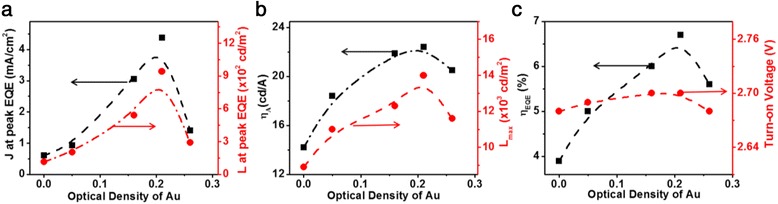
**a** The current density and luminance at peak EQE, **b** peak current efficiency and peak luminance (*L*_peak_), and **c** peak EQE and turn-on voltage characteristics of the QLEDs with varying Au NP concentrations in the PEDOT:PSS HIL

In Fig. 3 and Additional file 1: Figure S7, with the increase of the OD value of Au NPs, the current density and luminance at peak EQE, the peak luminance, current efficiency, and EQE all increase monotonically for Au NPs with OD ≤ 0.21 and all reach the maximum values at OD = 0.21, while all these performance parameters decrease monotonically for OD > 0.21. The peak luminance at OD = 0.21 is 14,000 cd/m^2^, corresponding to a growth of 55 % over that of the device without Au NPs (~9000 cd/m^2^). The maximum EQE and current efficiency increased from 3.9 % and 14.2 cd/A to 6.7 % and 22.4 cd/A, corresponding to improvements of 71 and 57 %, respectively, as compared with that of device without Au NPs. Such improvements in performance can be attributed to the more effective recombination of excitons in QLEDs with Au NPs. Additional file 1: Table S1 summarizes the relevant parameters of devices with Au NPs (12 nm in diameter, OD = 0, 0.05, 0.16, 0.21, 0.26). Furthermore, doping Au NPs slightly influences the turn-on voltage of QLEDs (Fig. 3c). These results prove that the optimized doping of Au NPs can significantly improve the performance of QLEDs due to the suppression of Auger recombination and the balanced electron-hole recombination.

**Fig. 4:**
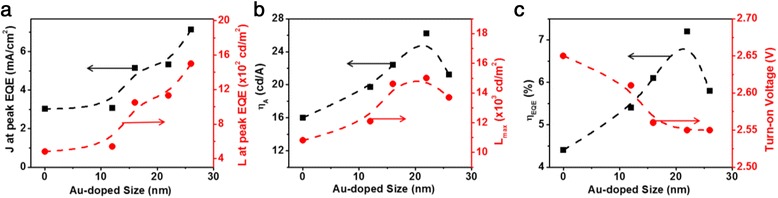
**a** The current density and luminance at peak EQE, **b** peak current efficiency and peak luminance (*L*_peak_), and **c** peak EQE and turn-on voltage characteristics of the QLEDs with varying Au NP dopant sizes in the PEDOT:PSS HIL

**Fig. 5:**
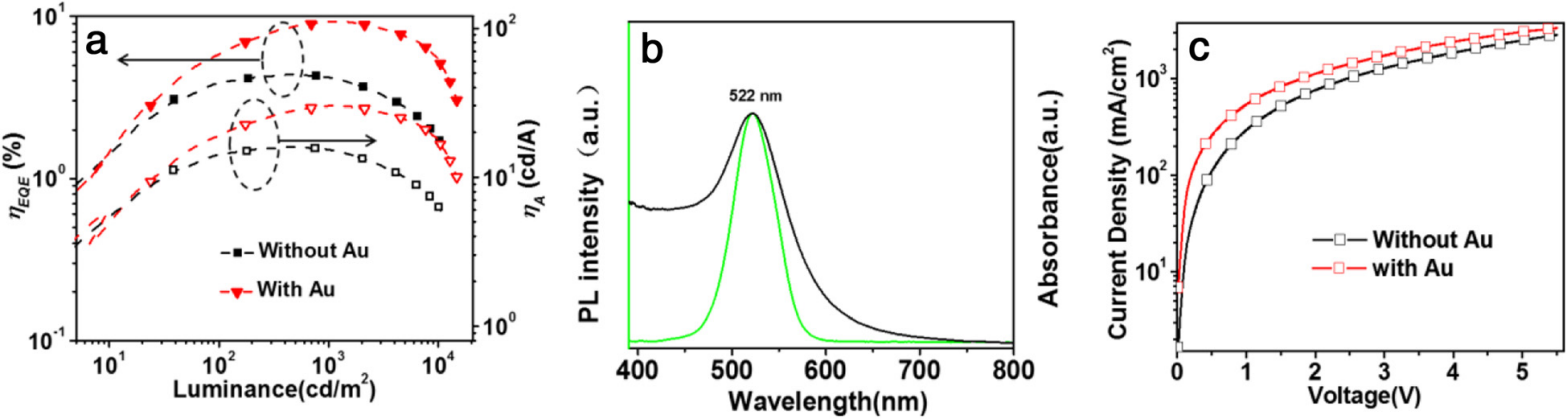
**a** EQE and current efficiency (*ƞ*_A_) versus luminance of optimized QLEDs based on HIL doped with and without Au NPs. **b** UV-Vis absorbance spectrum of 22-nm Au NPs in water and normalized PL spectrum of QDs in toluene **c** *J*-*V* curves of ITO/Au NP-doped PEDOT:PSS/Al and ITO/PEDOT:PSS/Al

Generally, the LSPR energy of Au NPs is not sensitive to their dimension [49, 50]. But here, we found that the LSPR effect is not only correlated with the concentration of Au NPs in PEDOT:PSS but also strongly associated with the size of Au NPs. PEDOT:PSS aqueous solutions contain four different-sized Au NPs (~12, ~16, ~22, ~26 nm) were respectively prepared with the same OD value of 0.21 and were then used as HIL materials. From Additional file 1: Figure S8, we can see that the maximum current density increases from 150 mA/cm^2^ (with unmodified PEDOT:PSS) to 168, 186, and 190 mA/cm^2^, corresponding to improvements of 12, 24, and 27 % for devices with Au NPs of 12, 16, and 22 nm, respectively. At the same time, as shown in Fig. 4b and Additional file 1: Figure S8b, a significant increase can be observed in the luminance after doping of Au NPs. For the devices with Au NPs of 12, 16, and 22 nm, the maximum values of luminance are improved from 10,800 cd/m^2^ for the control device to 12,100, 14,600, and 15,000 cd/m^2^, respectively. Figure 4b, c and Additional file 1: Figure S8c, d show the comparison of the current efficiency and EQE curves for QLEDs with different-sized Au NPs doped in HILs. It can be seen that EQE is improved from 4.4 % of PEDOT:PSS-only device to 5.4, 6.1, and 7.2 % for devices comprising dopants of 12-, 16-, and 22-nm-sized Au NPs in PEDOT:PSS HTLs. With a peak value of 26.2 cd/A, the current efficiency follows a similar trend with EQE. These results suggest that the carrier recombination of holes and electrons can be improved by optimization of doping different-sized Au NPs into the PEDOT:PSS layer. Thickness of QD layer is another key factor that affects the performance of QLEDs, and QLEDs with varying thicknesses of QD layer achieved by changing rotational speed are tested with fixed experimental conditions for other parameters (shown in Additional file 1: Figure S9 and Table S3). Twenty-two-nanometer-sized Au NPs are used as dopants and the end OD value of 0.21 for the doped PEDOT:PSS solution. The results indicate that the optimal thickness is ~27 nm, where the maximal EQE and current efficiency are 8.2 % and 29.1 cd/A (Fig. 5a, with Au), respectively, representing an improvement of 80 % over that of the control device without Au NPs (16 cd/A) (Fig. 5a).

In order to further understand why the QLEDs performance is enhanced by the introduction of Au NPs, the UV-Vis absorption spectrum of Au NPs in water and the normalized PL spectrum of ZnCdSe/ZnS core-shell QDs in toluene have been recorded, as shown in Fig. 5b. The LSPR peak of Au NPs (22 nm) is 522 nm, while the PL peak of ZnCdSe/ZnS core-shell QDs is 522 nm, indicating the good matching between the resonance frequency of LSPR and the emission band of the ZnCdSe/ZnS core-shell QDs [29]. When Au NPs were doped in PEDOT:PSS as HTL, the Au NPs will induce a strong enhancement of the local electromagnetic field intensity close to the NPs [51–53]. The electric field vector decays exponentially with distance away from the metal surface, with decay length of the order of one half of the excitation wavelength [54]. When ZnCdSe/ZnS core-shell QDs with PL peak at 522 nm is placed within the proper range of the enhanced local electric field, plasmonic interaction takes place, which can shorten the radiative lifetime of the QDs and enhance the radiative decay rates of the fluorescent species. As we know, the lifetime of excitons is defined as $\tau =\frac{1}{k_{\mathrm{r}}+{k}_{\mathrm{nr}}}$, where *k*_r_ is the radiative rate and *k*_nr_ is the non-radiative rate. The coupling between Au NPs and excitons results in an increase of *k*_r_ and has no effect on *k*_nr_, leading to a reduced lifetime of excitons in QDs as described in the main text. The quantum yield (*Q*) of QDs can be calculated by $Q=\frac{k_{\mathrm{r}}}{k_{\mathrm{r}}+{k}_{\mathrm{nr}}}$. The increased *k*_r_ must enhance the quantum yield (*Q*) of QDs, i.e., the electron-hole recombination in QDs is improved. A more efficient recombination means that more photons will be generated in the QDs, which improves the luminance of devices. For QLEDs with hybrid organic-inorganic CTLs discussed here, the electron mobility (ZnO as ETL, electron mobility ~2.0 × 10^−3^ cm^2^/V s) is usually much higher than the hole mobility (poly-TPD as HTL, electron mobility ~1.0 × 10^−4^ cm^2^/V s). The doping of Au NPs into HIL will help the hole injection and result in the balanced electron-hole recombination. In order to explore the influences of Au NPs on the hole transport ability of the PEDOT:PSS film, devices with structures of ITO/Au NP-doped PEDOT:PSS/Al and ITO/PEDOT:PSS/Al were fabricated. Figure 5c shows the *J*-*V* curves of these devices. The results indicate that the incorporation of Au NPs can significantly improve the hole current, that is to say that the doping of Au NPs is in favor of the hole injection, then leads to the balance of charge injection. The results demonstrate that Au NP as dopant also can offer a feasible and effective route for achieving high-performance QLEDs. In addition, we carried out SEM analysis on PEDOT:PSS films with and without Au NPs, as well as various layers of device. The SEM images (Additional file 1: Figure S10) show the continuous and homogeneous properties for all the films, indicating a negligible effect of Au dopant on the morphology of containing layers.

## Conclusions

In conclusion, the effect of Au NPs on the performance of QLEDs based on Au-doped PEDOT:PSS as HIL were carefully assessed. PEDOT:PSS with varying concentrations and sizes of Au NPs were adopted to determine the best doping conditions. The devices with the best performance were obtained with a 22-nm-sized Au NP dopant and an OD value of 0.21, of which the maximum EQE and current efficiency are 8.2 % and 29.1 cd/A, respectively, corresponding to 80 % improvement compared with that of the devices without Au NPs. The significant improvement may be attributed to the good electrical properties of Au NPs and the suppressed Auger recombination due to the enhanced exciton radiative rate in the emitting layer, which stems from the LSPR-induced near-field enhancement with the incorporation of the Au NP dopants. The results may be helpful to the acceleration of QD-based light-emitting applications like full-color displays and solid-state lighting.

## Additional file

Additional file 1: Details of synthesis of Zn_1–x_Cd_x_Se/ZnS core/shell QDs, ZnO NPs, TEM images of different-sized Au NPs in water, AFM images of different-sized Au NP-doped PEDOT:PSS films, PL decay curves of ZnCdSe/ZnS core-shell QDs film, characteristics of devices as a function of thickness of QDs layer, SEM images of PEDOT:PSS films without and with different concentrations Au NPs (OD = 0.21, 22 nm) as well as various layers of device. This information is available free of charge via the Internet or from the author. (DOCX 3760 kb)
