# Supplementary material for: Enhanced Performance of Quantum Dot-Based Light-Emitting Diodes with Gold Nanoparticle-Doped Hole Injection Layer
Source: Nanoscale Res Lett. 2016 Aug 24;11(1):376. doi: 10.1186/s11671-016-1573-8 (PMC4996815; doi:10.1186/s11671-016-1573-8)
Supplement: Additional file 1: — Details of synthesis of Zn1–xCdxSe/ZnS core/shell QDs, ZnO NPs, TEM images of different-sized Au NPs in water, AFM images of different-sized Au NP-doped PEDOT:PSS films, PL decay curves of ZnCdSe/ZnS core-shell QDs film, characteristics of devices as a function of thickness of QDs layer, SEM images of PEDOT:PSS films without and with different concentrations Au NPs (OD = 0.21, 22 nm) as well as various layers of device. This information is available free of charge via the Internet or from the author. (DOCX 3760 kb) [file 11671_2016_1573_MOESM1_ESM.docx]

**Additional file 1**

Enhanced performance of quantum dot-based light-emitting diodes with an Au nanoparticle doped hole-injection-layer

*Fei Chen,*^1^*^,^* ^2^*Qingli Lin,*^1^ *Hongzhe Wang,*^1^*^*^ Lei Wang,*^1^ *Fengjuan Zhang,*^1^ *Zuliang Du*^1^*^,^* ^2^ *Huaibin Shen,*^1^*^,^* ^2^*^*^ and Lin Song Li,*^1^*^*^*

^1^Key Laboratory for Special Functional Materials, Henan University, Kaifeng 475004, P. R. China

^2^Collaborative Innovation Center of Nano Functional Materials and Applications, Henan Province, P. R. China

*E-mail: [whz@henu.edu.cn](mailto:whz@henu.edu.cn); shenhuaibin@henu.edu.cn; lsli@henu.edu.cn

**Experimental Section**

**Chemicals.** All reagents were used as received without further experimental purification. Cadmium oxide (CdO, 99.99%), zinc oxide (ZnO, 99.9% powder), sulfur (S, 99.998%, powder), 1-octadecene (ODE, 90%), paraffin oil, oleic acid (OA, 90%), selenium (Se, 99.99%, powder), octadecylamine (ODA), 1-octanethiol (OT, 98%), zinc acetate (99.99 %), dimethyl sulphoxide (DMSO, 99.7%), tetramethylammonium hydroxide (TMAH, 97%), toluene (99%) and chlorobenzene (99%) were purchased from Aldrich. Hexanes (analytical grade) and methanol (analytical grade) were obtained from Beijing Chemical Reagent Ltd., China.

**Synthesis** **of** **Zn_1-x_Cd_x_Se/ZnS** **core/shell** **QDs** **with** **green** **emission**

**Preparation** **of** **precursors.** *Cd precursor*: A mixture of CdO (0.64 g, 5 mmol), oleic acid (5 mL, 20 mmol), and paraffin oil (45 mL ) was loaded into a 100 mL three-neck flask and heated to 240 ºC under nitrogen to obtain a colorless clear solution. *Zn precursor*: A mixture of ZnO (6.1 g, 75 mmol), oleic acid (210 mL, 750 mmol), and paraffin oil (165 mL) was loaded into a 500 mL flask and heated to 300 ºC under nitrogen to obtain a colorless clear solution. *Se precursor:* It was made by degassing Se (1.578 g, 20 mmol) and ODE (200 mL) in a 500 mL three-neck flask. The mixture was heated to 220 ºC under nitrogen, and then maintained for 180 min. Finally, a yellow color solution was obtained. *S precursor:* octanethiol diluted in ODE which 1.2 equivalent amounts refer to *Zn precursor*.

**Preparation of Zn_0.8_Cd_0.2_Se (Zn:Cd = 4:1) QDs.** 20 mL (2 mmol) of Se precursor and 50 mL of paraffin oil were loaded into a 250 mL flask and heated to 290 ºC under nitrogen flow. Then a mixture of Zn precursor (7.5 mL) and Cd precursor (5 mL) was injected into the flask, the reaction temperature was lowered to 260 ºC for QD growth. The as-synthesized QDs were purified by repeated precipitation with methanol and redispersion in hexanes for several times.

**Preparation of Zn_0.8_Cd_0.2_Se/ZnS core/shell QDs with green emission.** In a typical synthesis, 40 mL of ODE and 10 g of ODA were loaded into a 500 mL reaction vessel. The purified Zn_0.8_Cd_0.2_Se QDs (4.5 nm in diameter, 9.1×10^-7^ mol, dissolved in hexanes) were added into the mixture, and the system was kept at 100 ºC under nitrogen flow for 30 min to remove hexanes and other undesirable low vapor pressure materials. Subsequently, the reaction solution was heated to 310 °C under nitrogen flow and magnetic stirring, and a desired amount of Zn(OA)_2_ (10 mmol of ZnO mixed with 15 mL of oleic acid and 5 mL of paraffin was heated to 300 ^o^C to form a clear mixture solution under N_2_ flow.) and octanethiol (1.2 equivalent amounts refer to Zn(OA)_2_, diluted in 5 mL ODE) began to be injected dropwise into the reaction solution at a rate of 6 mL/h using a syringe pump. After finishing precursor infusion, the solution was further annealed at 310 ^o^C for 30 min. After the reaction was completed, the temperature was cooled down to room temperature and the QDs were purified using acetone or methanol.

**Preparation of ZnO NPs.** ZnO NPs were synthesized by a solution-precipitation process using zinc acetate and tetramethylammonium hydroxide (TMAH). According to the method previously reported, a solution of zinc acetate in dimethyl sulphoxide (DMSO) (0.5 M) and 30 mL of a solution of TMAH in ethanol (0.55 M) were mixed and stirred for 1 h in ambient air, then washed and dispersed in ethanol at a concentration of 30 mg/mL.

**Preparation of Au NPs.** A 100 mL sample of aqueous HAuCl_4_ (0.25 mM) was prepared in a 250 mL flask, containing controlled amounts of HCl or NaOH at room temperature. The solution was brought to boil while being stirred, and the corresponding amount of 5% aqueous sodium citrate with initial molar ratio of citrate to Au^3+^ was added. The reaction was allowed to run until the solution reached a wine red color, indicating the reaction was completed. The initial HAuCl_4_ concentration was varied from 0.125 to 1 mM. For the reactions in the series of variable Na_3_Ct/HAuCl_4_ ratios were performed by varying the concentration of Na_3_Ct with a fixed HAuCl_4_ concentration. The reactions in the series of variable pH were carried out by adding either HCl (1 M) or NaOH (1 M) into the reaction solution before the addition of Na_3_Ct.


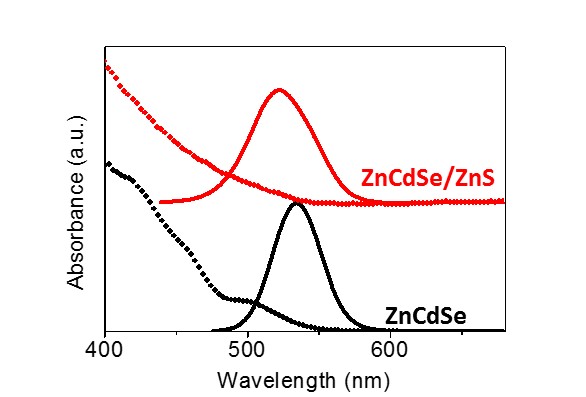


**Figure S1.** Evolution of absorption and PL spectra upon growth of ZnCdSe/ZnS core/shell QDs.


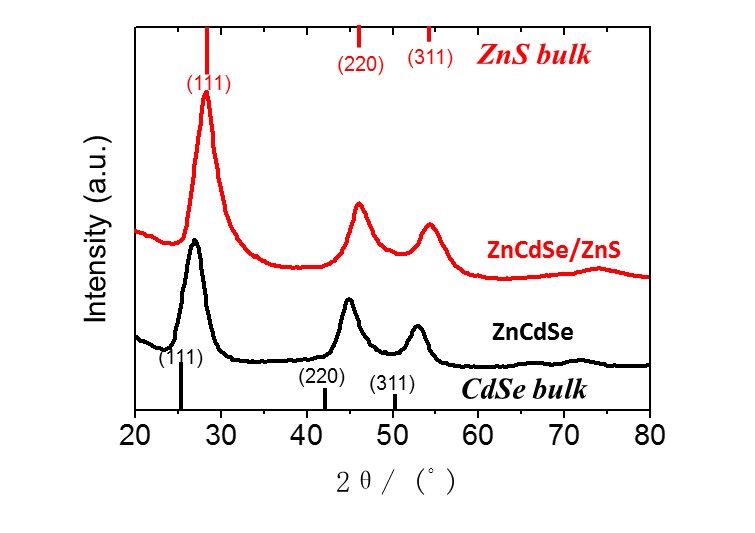


**Figure S2.** Powder XRD patterns of ZnCdSe cores and ZnCdSe/ZnS core/shell QDs.


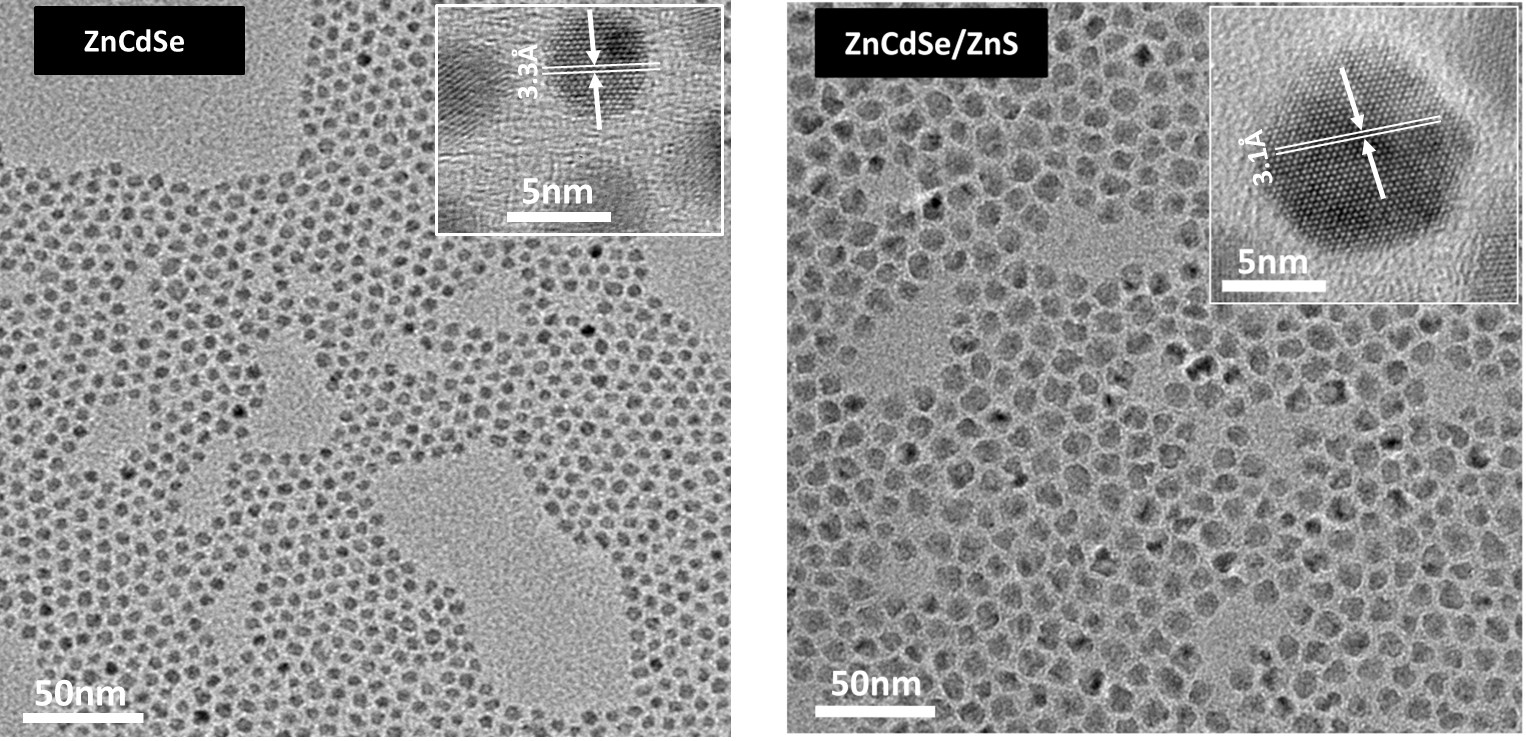


**Figure S3.** TEM and HRTEM images of ZnCdSe cores and ZnCdSe/ZnS core/shell QDs.


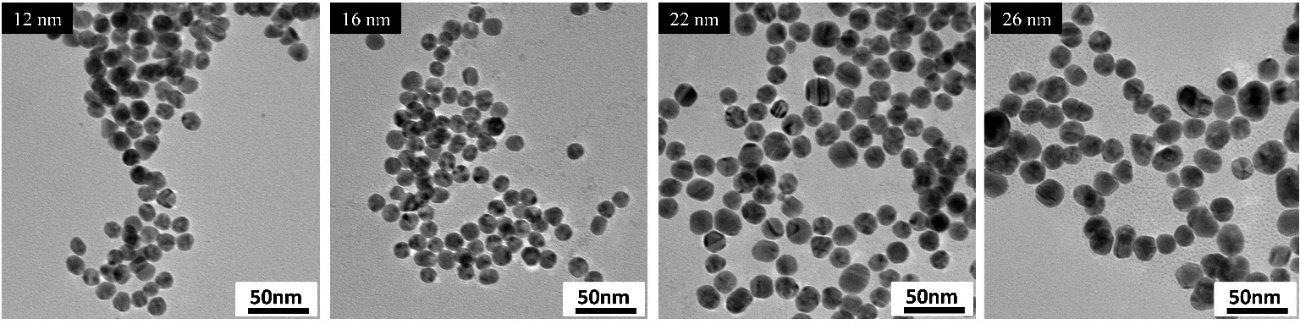


**Figure S4.** TEM images of different-sized Au NPs.


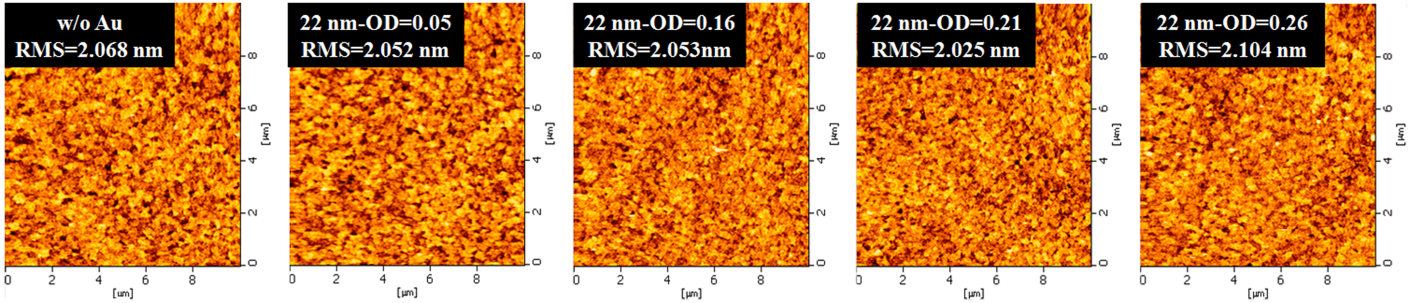


**Figure S5.** AFM images of different-sized Au NPs doped-PEDOT:PSS films.


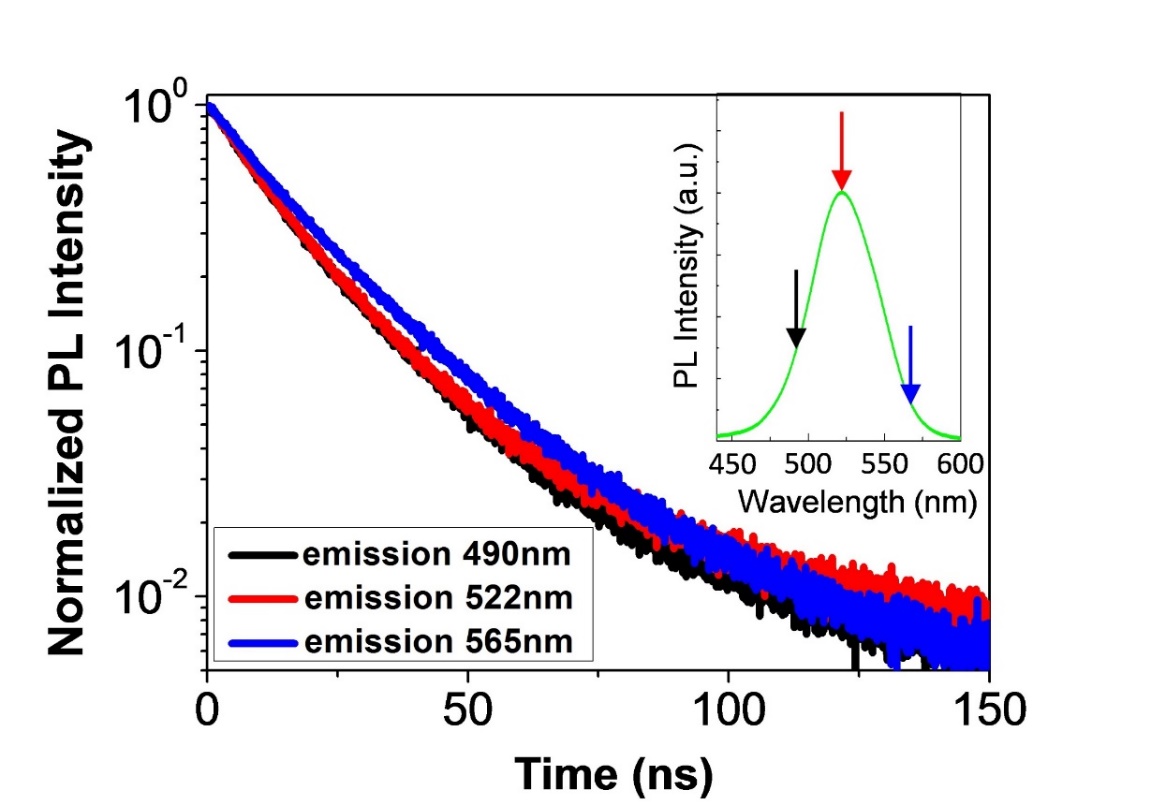


**Figure S6.** PL decay curves of ZnCdSe/ZnS core-shell QDs film samples collected at different emission wavelengths, *i.e.*, 490, 522, and 565 nm, which are also indicated with the arrows in the inset of PL spectrum.


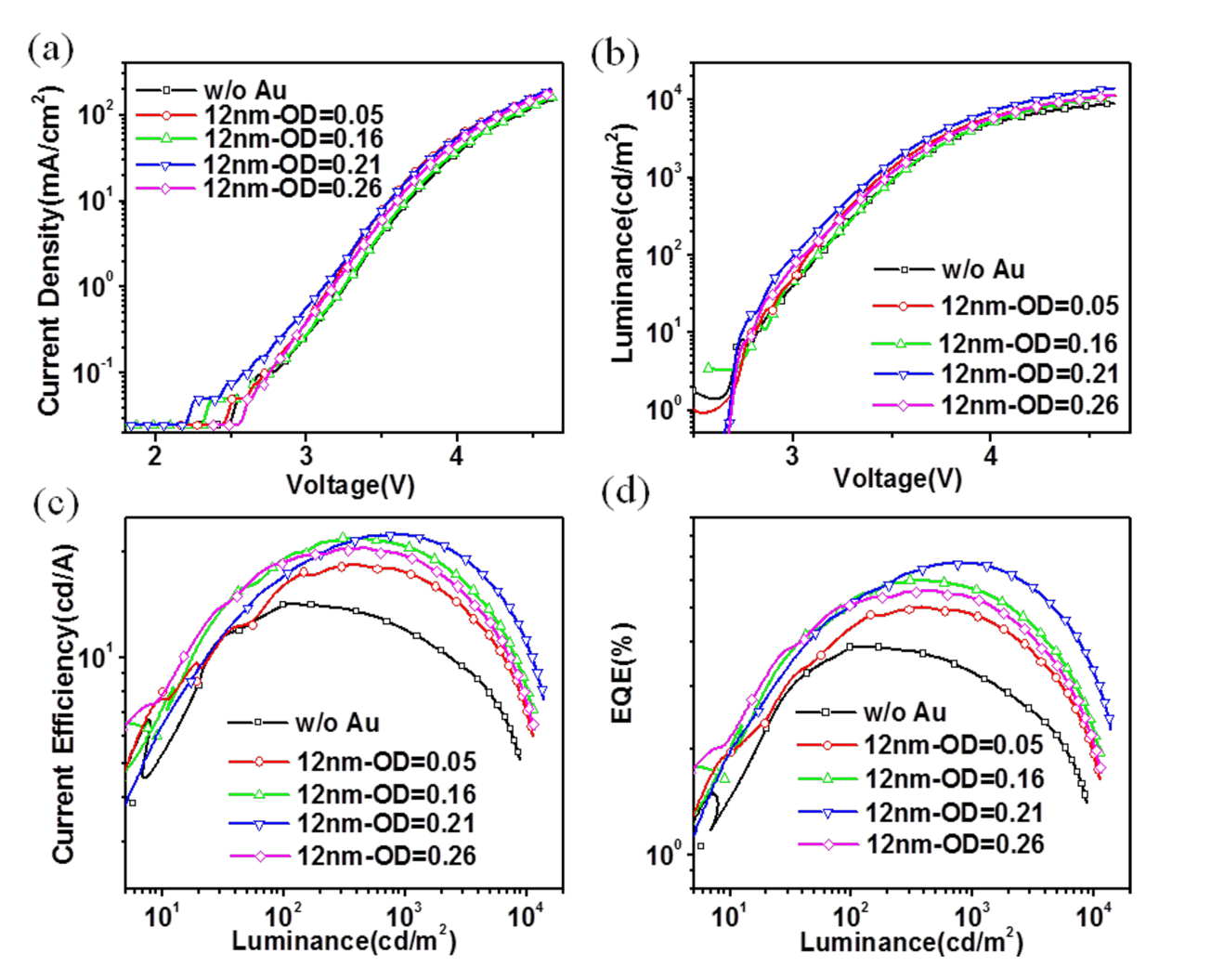


**Figure S7.** (a) The current density versus voltage (J-V), (b) luminance versus voltage (L-V), (c) current efficiency versus luminance (ƞ_A_-L ) and (d) EQE versus luminance (EQE-L) characteristics of the QLEDs with varying Au NP concentration in the PEDOT:PSS HIL.

**Table** S**1.** Summary of turn-on voltage, maximum luminance L_max_, maximum current efficiency ƞ_A_, and external quantum efficiency (EQE) of QLEDs with varying Au NP concentration in PEDOT:PSS.


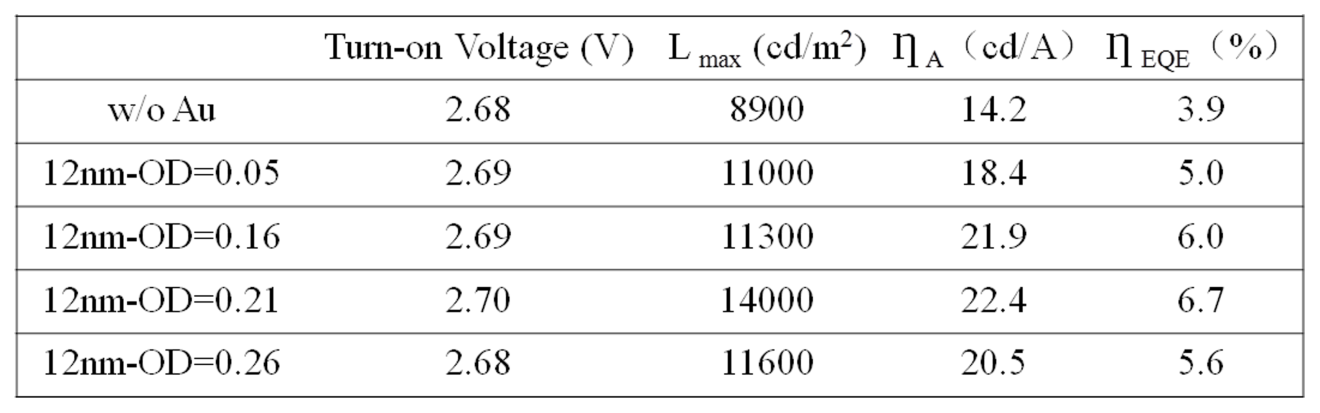


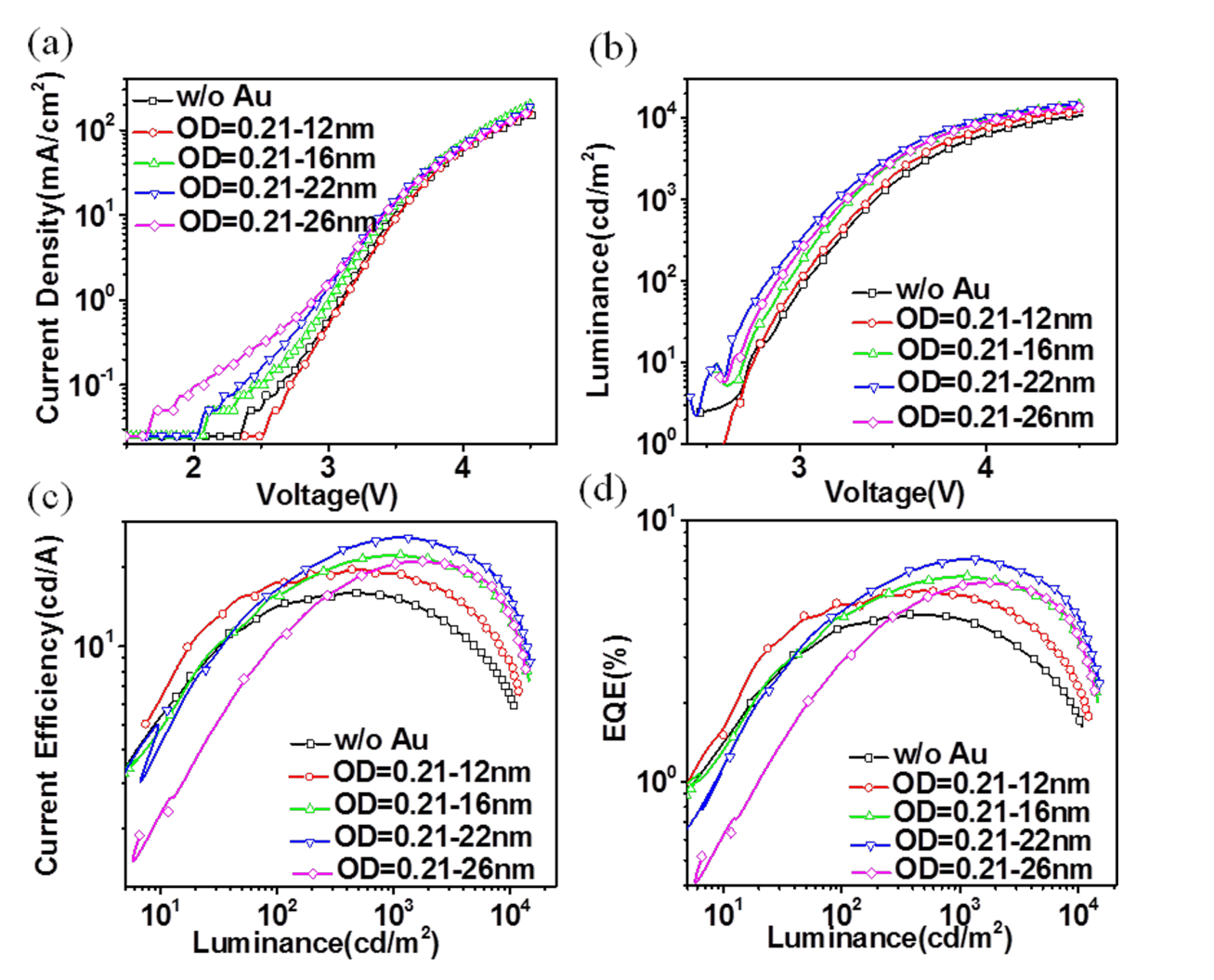


**Figure S8.** (a) The current density versus voltage (J-V), (b) luminance versus voltage (L-V), (c) current efficiency versus luminance (ƞ_A_-L) and (d) EQE versus luminance (EQE-L) characteristics of the QLEDs with varying dimension of Au NP dopants in the PEDOT:PSS HIL.

**Table** S**2.** Summary of turn-on voltage, maximum luminance L_max_, maximum current efficiency ƞ_A_, and EQE of QLEDs with different-sized Au NPs in PEDOT:PSS.


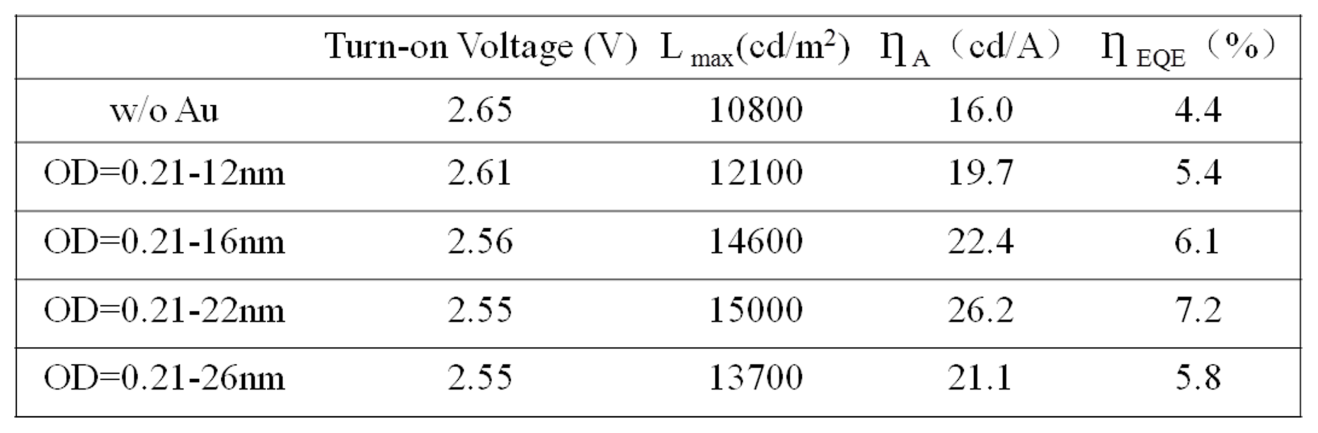


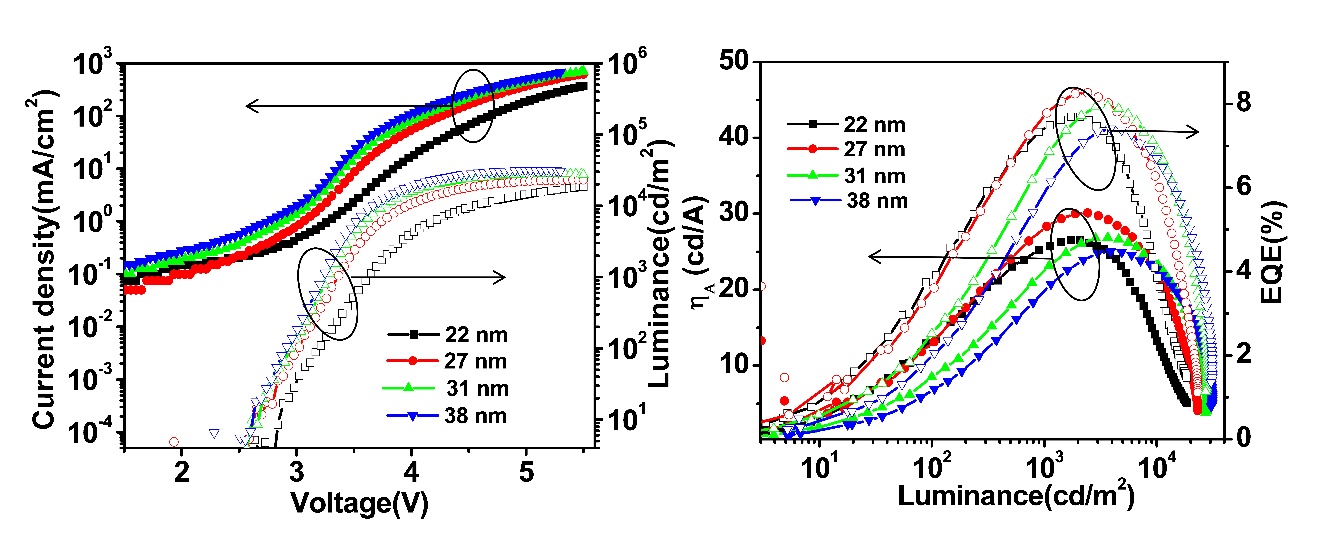


**Figure S9.** (a) Current density (J) and luminance (L) of the devices based on PEDOT:PSS with 22 nm Au NPs at the OD=0.21 as a function of driving voltage (V). (b) External quantum efficiency (EQE) and current efficiency (ƞ_A_) as a function of luminance.

**Table S3.** Summary of maximum current efficiency and EQE of QLEDs with varying thickness of QDs.


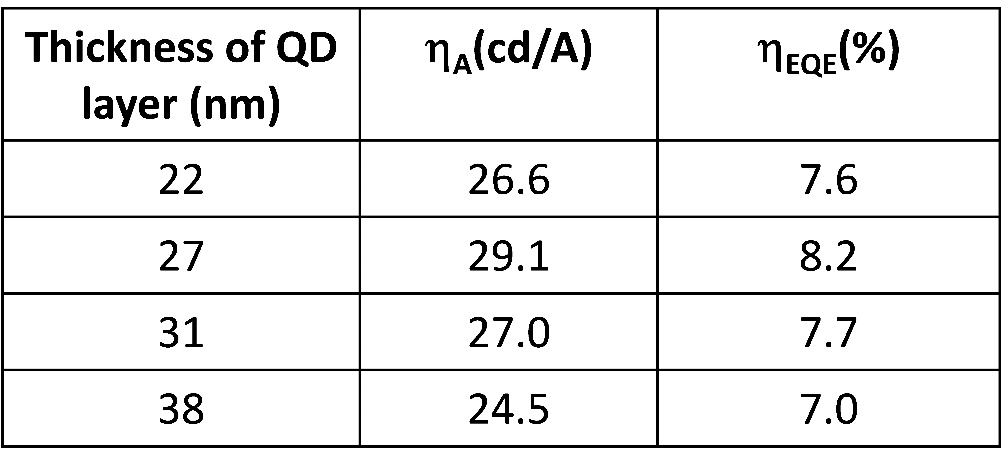


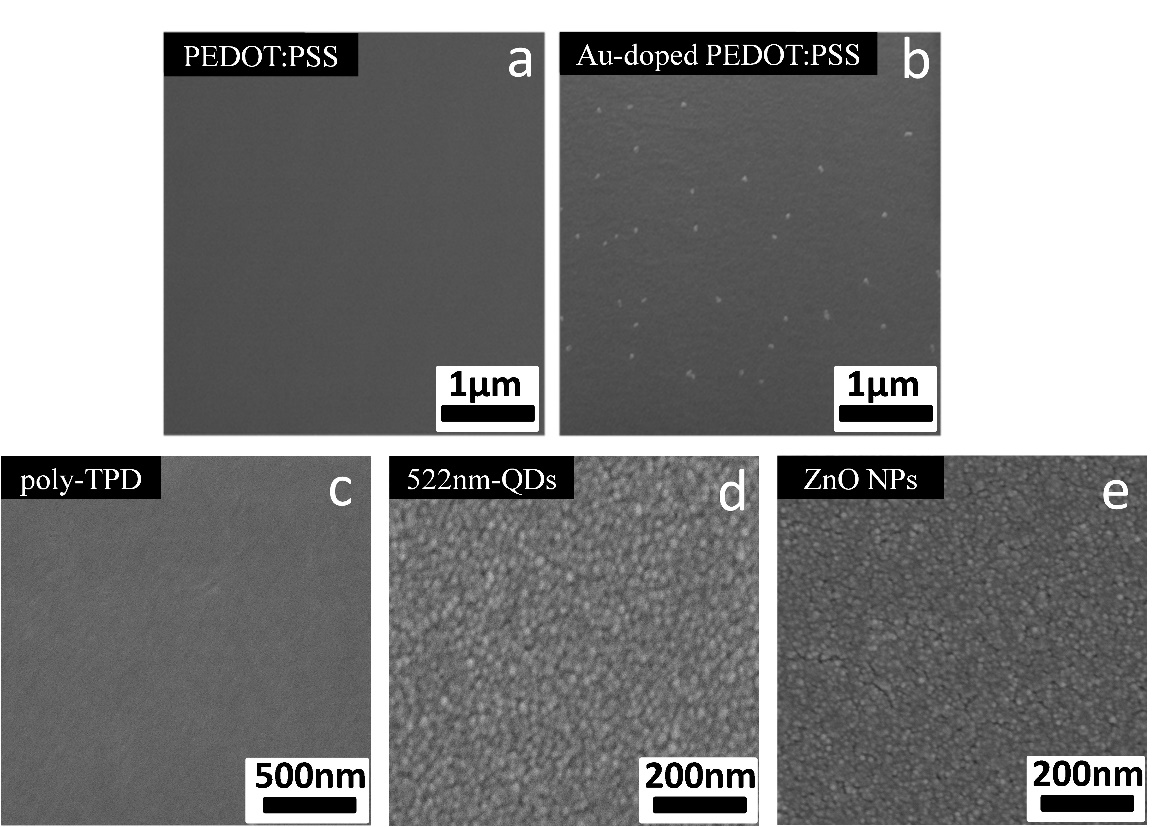


**Figure S10.** SEM images of PEDOT:PSS films without and with different concentrations Au NPs (OD=0.21, d=22nm) as well as various layers of device (a: ITO/PEDOT:PSS, b: ITO/Au-PEDOT:PSS, c: ITO/Au-PEDOT:PSS/poly-TPD, d: ITO/Au-PEDOT:PSS/poly-TPD/QDs, e: ITO/Au-PEDOT:PSS/poly-TPD/QDs/ZnO).
